# The impact of cataract surgery on vision-related quality of life for bilateral cataract patients in Ho Chi Minh City, Vietnam: a prospective study

**DOI:** 10.1186/1477-7525-12-16

**Published:** 2014-02-06

**Authors:** Kien Gia To, Lynn B Meuleners, Michelle L Fraser, Dung Van Do, Dat Van Duong, Van-Anh Ngoc Huynh, Quyen Gia To, Tien Duy Phi, Hoang Huy Tran, Nguyen Do Nguyen

**Affiliations:** 1 University of Medicine and Pharmacy, Ho Chi Minh City, Vietnam; 2 Curtin-Monash Accident Research Centre (C-MARC), Curtin University, Perth, Australia; 3 United Nations Population Fund, Hanoi, Vietnam; 4 The Eye Hospital, Ho Chi Minh City, Vietnam; 5 University of South Carolina, Columbia, USA

**Keywords:** Vision, Public health, Epidemiology, Cataract, Quality of life

## Abstract

**Background:**

To determine the impact of cataract surgery on vision-related quality of life (VRQOL) and examine the association between objective visual measures and change in VRQOL after surgery among bilateral cataract patients in Ho Chi Minh City, Vietnam.

**Methods:**

A cohort of older patients with bilateral cataract was assessed one week before and one to three months after first eye or both eye cataract surgery. Visual measures including visual acuity, contrast sensitivity and stereopsis were obtained. Vision-related quality of life was assessed using the NEI VFQ-25. Descriptive analyses and a generalized linear estimating equation (GEE) analysis were undertaken to measure change in VRQOL after surgery.

**Results:**

Four hundred and thirteen patients were assessed before cataract surgery and 247 completed the follow-up assessment one to three months after first or both eye cataract surgery. Overall, VRQOL significantly improved after cataract surgery (p < 0.001) particularly after both eye surgeries. Binocular contrast sensitivity (p < 0.001) and stereopsis (p < 0.001) were also associated with change in VRQOL after cataract surgery. Visual acuity was not associated with VRQOL.

**Conclusions:**

Cataract surgery significantly improved VRQOL among bilateral cataract patients in Vietnam. Contrast sensitivity as well as stereopsis, rather than visual acuity significantly affected VRQOL after cataract surgery.

## Background

Vietnam is a developing country with a population of 85.9 million people, of which 17% are aged 50 years or older [1]. The incidence rate of cataract was estimated at 170,000 cases per year [2]. The increasing prevalence of cataract, its potential to cause severe visual disability and its impact on quality of life [3] means that a firm understanding of the impact of cataract surgery on visual and other outcomes is important for policy development.

Strong evidence exists that cataract surgery significantly improves vision-related quality of life (VRQOL) [4-6]. However, the majority of research has been conducted in developed countries where visual impairment prior to cataract surgery is less severe and social circumstances differ considerably [7]. A recent study undertaken in rural Vietnam examined quality of life following cataract surgery [7]. However this study was cross-sectional, therefore change in quality of life after surgery could not be assessed.

It is currently unclear which measures of vision are associated with improvement in VRQOL after cataract surgery. Cataract can affect several different aspects of vision with visual acuity being the traditional measure used to assess impairment. However, a prospective study from Spain found that the influence of visual measures on VRQOL changed throughout the different stages of cataract surgery [8]. Before first eye surgery binocular visual acuity was strongly associated with VRQOL. However, after first and second eye surgery stereopsis was most strongly associated with VRQOL, followed by binocular contrast sensitivity, with visual acuity showing only a weak association [8]. Similarly, a study in the UK reported that binocular visual acuity was the measure most strongly associated with VRQOL before surgery [9]. However change in VRQOL after surgery was found to be strongly associated with change in stereopsis, followed by binocular contrast sensitivity and least associated with change in visual acuity [9]. A low correlation between change in visual acuity and VRQOL was also found in a study from Finland [10]. The only conflicting results were reported from a study which found that change in visual acuity and disability glare but not contrast sensitivity were associated with change in VRQOL [11]. Stereopsis was not examined in this study.

To date, most studies have used the Visual Function Index (VF-14) to measure VRQOL among cataract patients and the majority were conducted in developed countries [12]. This instrument has been criticised for not addressing all the visual concerns of cataract patients [13], being highly focused on activities that require visual acuity [9] and having ceiling effects [8,9]. The National Eye Institute Visual Function Questionnaire (NEI VFQ-25) addresses a wider range of visual concerns, including social and mental outcomes of visual impairment [14] and allows investigation into which specific aspects of VRQOL improve after cataract surgery.

Therefore this study aims to determine the impact of cataract surgery on VRQOL and examine the association between objective visual measures and change in VRQOL after surgery among bilateral cataract patients in Ho Chi Minh City, Vietnam.

## Methods

### Study design and sample

A prospective cohort study using a before and after design was undertaken. Inclusion criteria for participants were: age-related bilateral cataract; scheduled to undergo first eye or first and second eye cataract surgery; living independently in the community and aged 50 years or older. Exclusion criteria were: previous cataract surgery; injury or diabetes-related cataract, a diagnosis of glaucoma or any other significant ocular conditions, a diagnosis of dementia, Parkinson’s disease, schizophrenia or being wheelchair bound.

### Data collection

Participants were recruited between July 2011 and July 2012. Before any data was collected, informed written consent was obtained from each participant. Participation was voluntary and patients were informed they could withdraw from the study at any time without consequence.

Data was collected at two time points at least a week before first eye cataract surgery and one to three months after either first eye surgery or both first and second eye surgery. Information was collected using a researcher-administered questionnaire and three objective visual tests. All cataract surgeries were undertaken by phacoemulsification.

### Questionnaire

Demographic data and information on current prescribed medications, refractive lenses worn, and co-morbid conditions were collected via a researcher-administered questionnaire.

The NEI VFQ-25 was used to examine the impact of visual impairment on vision-related quality of life. This questionnaire has been previously validated for use with cataract patients [14]. The basis and design of this questionnaire have been described in full elsewhere [14]. The NEI VFQ-25 was translated into Vietnamese and back translated into English by two independent translators. The “driving” subscale of the NEI VFQ-25 was replaced with a “riding” subscale as motorcycle/moped riding is more common in Vietnam than driving.

#### Objective visual measures

Three objective measures of vision were administered by the researcher according to hospital protocol. Participants wore their current corrective lenses for visual testing. Binocular visual acuity (with both eyes) was measured using a Snellen chart [15,16]. Scores were expressed on a logarithm of the minimum angle of resolution (logMAR) scale. Binocular contrast sensitivity was measured using a Pelli-Robson chart in log units [17]. Stereopsis, a form of depth perception, was assessed by the Titmus Fly Stereotest (Stereo Optical Co., Inc.) which measured disparity from 1.602 to 3.551 log seconds of arc.

### Statistical analysis

Descriptive analyses were undertaken. Inferential statistics were used to compare visual and vision-related quality of life variables before and after surgery.

A generalised linear estimating equations (GEE) model was undertaken to examine the impact of cataract surgery on the VRQOL composite score. The GEE model was fitted for the 247 participants for whom complete before and after surgery data were available. Time (before or after surgery) was added to the model as an explanatory variable.

Binocular measures of visual acuity and contrast sensitivity as well as stereopsis were included as explanatory variables in the model. In addition, potential confounders included in the model were age, gender, education level, employment status, marital status, living situation (alone: no/yes), ethnicity, taking prescription medications (no/yes), co-morbid medical conditions including other eye conditions (no/yes) and wears glasses after surgery (no/yes). A variable was also added to specify whether a participant had first eye cataract surgery only or both first and second eye cataract surgery during the follow-up period. All statistical analyses were performed using STATA version 10.

#### Ethical issues

The study was approved by the Curtin University Human Research Ethics Committee (approval number HR68/2011) and the executive board of the Eye Hospital in Ho Chi Minh City, Vietnam.

## Results

Four hundred and thirty four bilateral cataract patients were approached to participate in the study, of which 10 refused and 11 did not meet the inclusion criteria for the study. The final sample consisted of 413 patients, resulting in a response rate of 95.1%. Approximately 40% of participants were lost to follow-up with 247 participants completing the second assessment. There were no significant differences between those who completed both assessments and those who completed the baseline assessment only in terms of gender, age, ethnicity, marital status, living alone, education level, co-morbid conditions or baseline NEI VFQ-25 composite scores. However, a significantly greater proportion of participants who completed the study wore glasses (p = 0.01) and took prescription medication (p = 0.03). Among participants who completed both assessments, 43.3% (n = 107) of patients had first eye cataract surgery only and 56.7% (n = 140) had both first and second eye cataract surgery during the study period.

Table 1 compares participants who completed the baseline assessment (n = 413) and those who completed the baseline and follow up assessment (n = 247) by those who had first eye surgery (n = 107) and those who had first and second eye surgery (n = 140). The majority of participants who had first eye only and both eye surgeries were female (56% and 67.9% respectively); aged between 60-69 years (44.9% and 38.6%); were married (64.5% and 57.9%); had a co-morbidity (60.8% and 61.4%) and did not take any medications (57% and 60%).

**Table 1:** Demographic characteristics of participants who completed baseline (n = 413) and follow-up assessments (n = 247)

**Variables**	**Baseline assessment (n = 413)**		**Follow-up assessment (n = 247)**				**p-value**
			**First eye surgery only (n = 107)**		**Both eye surgeries (n = 140)**		
	**N**	**%**	**N**	**%**	**N**	**%**	
Gender							
Female	268	64.9	60	56.1	95	67.9	0.99
Male	145	35.1	47	43.9	45	32.1	
Age (years)							
50-59	86	20.8	28	26.2	30	21.4	0.98
60-69	170	41.2	48	44.9	54	38.6	
70+	157	38.0	31	29.0	56	40.0	
Marital status							
Married	239	57.9	69	64.5	81	57.9	0.95
Single/widow/divorced	174	42.1	38	35.5	59	42.1	
Ethnicity							
Kinh	401	97.1	102	95.3	136	97.1	0.97
Other	12	2.9	5	4.7	4	2.9	
Live alone							
No	383	92.7	99	92.5	134	95.7	0.84
Yes	30	7.3	8	7.5	6	4.3	
Education level							
Junior high school	303	73.4	79	73.8	99	70.7	0.96
Senior high school	110	26.6	28	26.2	41	29.3	
Currently employed							
No	326	78.9	78	72.9	111	79.3	0.92
Yes	87	21.1	29	27.1	29	20.7	
Wears glasses							
No	298	72.2	82	76.6	88	62.9	0.70
Yes	115	27.8	25	23.4	52	37.1	
Takes prescription medication							
No	250	60.5	61	57.0	84	60.0	0.59
Yes	163	39.5	46	43.0	56	40.0	
Co-morbid condition							
No	145	35.1	42	39.3	54	38.6	0.13
Yes	268	64.9	65	60.8	86	61.4	

Patient’s visual test scores before and after cataract surgery are presented in Table 2. Before surgery, participants had a mean binocular visual acuity of 0.58 logMAR (6/23) (SD = 0.38). Among participants who had first eye surgery only (n = 107), mean binocular visual acuity improved by 0.42 logMAR to 0.16 logMAR (6/9) (SD = 0.23). Among participants who underwent both first and second eye surgery (n = 140), mean binocular visual acuity improved by 0.54 logMAR to 0.04 logMAR (6/7) (SD = 0.01). This equated to a clinically meaningful improvement of 21 letters or 4.2 lines on the ETDRS chart for first eye only patients and 27 letters or 5.4 lines for patients who had both eyes operated on. Clinicians often define a change of 0.1 logMAR units or one line on the chart as clinically meaningful [18].

**Table 2:** Mean visual test scores before and after cataract surgery (n = 247)

	**Before surgery (n = 247) Mean (SD)**	**After surgery Mean (SD)**		p-value**
		**First eye surgery only (n = 107)**	**First and second eye surgery (n = 140)**	
Binocular visual acuity (logMAR units)^*^	0.58 (0.38)	0.16 (0.23)	0.04 (0.12)	<0.001
Binocular contrast sensitivity (log units)	1.13 (0.43)	1.69 (0.32)	1.85 (0.16)	<0.001
Binocular stereopsis (log seconds of arc)^*^	2.71 (0.70)	2.38 (0.62)	1.96 (0.33)	<0.001

logMAR: logarithm of the minimum angle of resolution, SD: standard deviation.

*Lower scores represent better vision.

**from ANOVA.

Mean binocular contrast sensitivity before surgery was 1.13 log units (SD: 0.02). Among participants who had first eye surgery only, mean binocular contrast sensitivity improved by 0.56 log units to 1.69 log units (SD = 0.03). Among participants who underwent both first and second eye surgery, mean binocular contrast sensitivity improved by 0.72 log units to 1.85 log units (SD = 0.01). This translated to a clinically significant improvement of over 11 letters or 1.9 lines on the Pelli-Robson chart for patients who underwent first eye surgery only and over 14 letters or 2.4 lines for those who underwent first and second eye surgery. A change of 0.3 log units or one line is often considered to be clinically meaningful [19].

Mean stereopsis before surgery was 2.71 log seconds of arc (SD = 0.70). For participants who underwent first eye cataract surgery only, stereopsis significantly improved by 0.33 log seconds to 2.38 log seconds of arc (SD: 0.62). For participants who underwent first and second eye surgery, stereopsis improved 0.77 log seconds to 1.96 log second of arc (SD = 0.33). A change in stereopsis of 0.30 log seconds of arc is often defined as clinically meaningful [20].

Composite and subscale VRQOL scores for the NEI-VFQ-25 before and after cataract surgery are presented in Table 3. Before surgery, the mean composite VRQOL score was 65.19 points (SD = 16.80). No participant scored the maximum possible composite score of 100.00. For those who had first eye cataract surgery only, composite scores improved on average by 22.83 points to a mean score of 88.02 (SD = 14.51). For those who had first and second eye surgery, composite scores improved by 29.32 points to 94.51 points (SD = 4.94). This change was statistically significant (p < 0.001) and clinically meaningful as approximately a six point change in composite score has been considered to be meaningful [21].

**Table 3:** Vision-related quality of life scores before and after cataract surgery (n = 247)

**NEI-VFQ-25 Subscale**	**Before surgery (n = 247) Mean (SD)**	**After surgery Mean (SD)**		p-value**
		**First eye surgery only (n = 107)**	**First and second eye surgery (n = 140)**	
General health	30.97 (14.34)	32.94 (18.04)	34.11 (16.21)	0.04
General vision	39.43 (11.32)	62.06 (17.14)	75.29 (10.35)	p < 0.001
Ocular pain	75.35 (30.69)	84.58 (25.09)	85.00 (23.95)	0.03
Near activities	64.74 (23.27)	86.25 (22.20)	95.42 (9.15)	p < 0.001
Distance activities	70.48 (24.09)	92.06 (17.82)	98.36 (5.86)	p < 0.001
Vision specific				
Social functioning	86.34 (19.86)	96.50 (13.61)	100 (0)	p < 0.001
Mental health	63.11 (28.90)	87.62 (19.58)	95.22 (11.01)	p < 0.001
Role difficulties	42.46 (46.48)	88.90 (30.44)	98.04 (12.39)	p < 0.001
Dependency	78.24 (28.94)	96.73 (9.76)	99.17 (4.93)	p < 0.001
Motorcycle/ moped riding*	51.47 (34.66)	86.86 (28.25)	92.86 (20.50)	p < 0.001
Color vision	94.23 (12.74)	98.26 (11.01)	100 (0)	p < 0.001
Peripheral vision	45.34 (26.55)	89.49 (23.05)	99.29 (4.18)	p < 0.001
Composite score	65.19 (16.80)	88.02 (14.51)	94.51 (4.94)	p < 0.001

*missing information.

**from ANOVA.

VRQOL subscale scores before surgery were lowest for general health with a mean score of 30.97 (SD = 14.34), general vision (mean = 39.43; SD = 11.32), vision specific role difficulties (mean = 42.46; SD = 46.48) and peripheral vision (mean = 45.34; SD = 26.55). Scores were highest for colour vision (mean = 94.23; SD = 12.74) and vision specific social functioning (mean = 86.34; SD = 19.86). There were statistically significant improvements in mean scores for the all the VRQOL subscales after surgery for participants who had first eye surgery only, as well as participants who had both first and second eye surgery. However, all subscale scores were higher after surgery for those who had both eyes operated, compared to those who had first eye surgery only.

The results of the adjusted multivariate GEE linear regression model examining change in composite NEI VFQ-25 score after cataract surgery are presented in Table 4. After adjusting for potential confounders there was a significant improvement of nearly 15 points in the VRQOL composite score after cataract surgery (p < 0.001). In addition, those who had first and second eye cataract surgery reported a significant improvement in VRQOL of nearly 4 points (p < 0.001) compared to those who had first eye surgery only. Binocular contrast sensitivity and stereopsis were both significantly associated with improvement in VRQOL after surgery, however, visual acuity was not. For every one log unit increase in contrast sensitivity (better vision), VRQOL score also improved by over 13 points (p < 0.001). Finally, for every log second increase in stereopsis (poorer vision), VRQOL score decreased by over three points (p < 0.001).

**Table 4:** Generalised linear estimating equation model of vision-related quality of life after cataract surgery (n = 247)

**Variable**	**Coefficient (SE)**	**95% confidence interval**	**pcvalue**
Before or after surgery: after	14.87 (1.47)	11.98 to 17.75	<0.001*
Surgery: both eyes	3.66 (1.28)	1.13 to 6.19	0.004*
Binocular visual acuity (logMAR)	-2.06 (2.72)	-7.39 to 3.27	0.45
Binocular contrast sensitivity (log units)	13.27 (2.37)	8.62 to 17.92	<0.001*
Stereopsis (log seconds of arc)	-3.31 (0.99)	-5.29 to-1.33	<0.001*
Age (years)	0.06 (0.08)	-0.11 to 0.23	0.46
Gender: female	2.76 (1.53)	-0.23 to 5.76	0.07
Ethnicity: other	-1.01 (3.33)	-7.54 to 5.51	0.76
Lives alone: yes	-0.71 (2.90)	-6.40 to 5.00	0.81
Married: yes	-2.96 (1.52)	-5.94 to 0.21	0.05
Co-morbidity: yes	-2.33 (1.55)	-5.38 to 0.73	0.14
Prescribed medication: yes	2.25 (1.54)	-0.77 to 5.27	0.14
Employed: yes	2.96 (1.61)	-0.20 to 6.13	0.06
Education: > junior high school	1.91 (1.47)	-0.97 to 4.80	0.19
Wears glasses after surgery: yes	1.02 (1.26)	-1.45 to 3.51	0.41

## Discussion

This is one of the first studies to examine the association between visual function and VRQOL before and after cataract surgery for an ageing population in Vietnam. There was a significant improvement of nearly 15 points in the VRQOL composite score after cataract surgery after controlling for confounding factors. These results are consistent with previous research examining change in NEI VFQ-25 scores after cataract surgery in Japan and the USA [22,23].

A strength of the NEI VFQ-25 questionnaire is that it not only measures difficulty with visual tasks but also the influence of visual impairment on social functioning, mental health, role difficulties and dependency [14]. While large improvements were seen in subscales such as general vision and near activities, they were also seen in the vision specific mental health and role difficulties subscales for those who had first eye surgery and surgery in both eyes. This suggests that cataract surgery has benefits not only for daily activities but also has wide reaching social and mental health benefits. It should be noted however, that the NEI VFQ-25 has been criticised for its lack of unidimensionality as it measures a combination of visual functioning and socioemotional constructs [24]. This may decrease the validity of the composite score.

The results also confirmed that while bilateral cataract patients who had only first eye surgery experienced improvement in VRQOL, those who had both eyes operated on experienced a significantly greater improvement of nearly four points on the NEI-VFQ-25. They also scored better on each of the subscales after surgery, compared to those who had first eye surgery only. This finding highlights the importance of recommending second eye surgery to bilateral cataract patients.

A strength of this study was the inclusion of the three objective visual measures. Previous studies examining the impact of cataract surgery on VRQOL have seldom measured stereopsis, which was significantly associated with improved VRQOL. A further strength of the study is that it also examined the impact of cataract surgery on VRQOL for bilateral cataract patients who had only first eye surgery compared to those who had both eyes operated on. First-eye surgery has been shown to bring about significant improvements in visual acuity, contrast sensitivity and stereopsis. The results of this study confirm these findings with significant clinical improvements found after cataract surgery for binocular measures of vision despite mean baseline vision of participants in our study being worse than that of participants in studies from developed countries such as Spain and Denmark [4,8,25].

Improvement in contrast sensitivity and stereopsis were associated with an improvement in VRQOL after surgery, however visual acuity was not. This finding is consistent with previous research [8,9]. Visual acuity is the most commonly used measure for assessing visual impairment and prioritising cataract patients for surgery. These findings indicate that contrast sensitivity and stereopsis may also be important measures to consider when determining the level of impairment caused by cataract. Contrast sensitivity is important for many everyday activities and the performance of tasks in low light, as the environment contains several low contrast stimuli [9,26,27]. Therefore, it is likely that contrast sensitivity is important for VRQOL. A UK-based trial of first eye cataract surgery also reported that change in VRQOL after surgery was most strongly related to change in stereopsis, followed by contrast sensitivity [9]. Further research is required to elucidate the mechanism by which stereopsis may impact on quality of life outcomes. Finally, the study is strengthened by the adjustment for other factors which are well known confounders of vision-related quality of life in an ageing population, such as medication usage, co-morbidities including other ocular conditions and wearing glasses.

The lack of a suitable comparison group that had cataract, but did not have surgery was a limitation of the study. However the “before and after” design using the same person as their own control would account for other inter-individual differences that can confound studies using separate control groups. Cognitive ability was also not captured and is a well-known confounder in research with an older population. In addition, there was a high drop-out rate for the study (40%). This is because many participants were unable to return to the hospital for their follow-up assessment.

## Conclusions

In conclusion, this study has provided valuable information about change in VRQOL after surgery in Vietnam. Cataract surgery significantly improved VRQOL among bilateral cataract patients in Vietnam. Contrast sensitivity as well as stereopsis, rather than visual acuity significantly affected VRQOL after cataract surgery. Due to the benefits that cataract surgery has for VRQOL, ensuring that the older population has access to regular eye examinations and timely treatment for cataract is paramount.

## Competing interests

The authors declare that they have no competing interests.

## Authors’ contributions

KGT, LBM and MLF contributed to the study design, data analysis, interpretation of results and preparation of the manuscript. KGT also conducted and managed the study. DVD and DVD contributed to the design and management of the study and reviewed the manuscript. VNH, QGT, TDP, HHT and NDN provided management for the study and reviewed the manuscript. QGT also assisted with data analysis. All authors read and approved the final manuscript.
